# Alanine Aminotransferase Decreases with Age: The Rancho Bernardo Study

**DOI:** 10.1371/journal.pone.0014254

**Published:** 2010-12-08

**Authors:** Mamie H. Dong, Ricki Bettencourt, Elizabeth Barrett-Connor, Rohit Loomba

**Affiliations:** 1 Division of Gastroenterology, Department of Medicine, University of California San Diego, La Jolla, California, United States of America; 2 Division of Epidemiology, Department of Family and Preventive Medicine, University of California San Diego, La Jolla, California, United States of America; The University of Hong Kong, Hong Kong

## Abstract

**Background:**

Serum alanine aminotransferase (ALT) is a marker of liver injury. The 2005 American Gastroenterology Association Future Trends Committee report states that serum ALT levels remain constant with age. This study examines the association between serum ALT and age in a community-dwelling cohort in the United States.

**Methods:**

A cross-sectional study of 2,364 (54% female) participants aged 30–93 years from the Rancho Bernardo Study cohort who attended a research clinic visit in 1984–87. Demographic, metabolic co-variates, ALT, bilirubin, gamma glutamyl transferase (GGT), albumin, and adiposity signaling biomarkers (leptin, IL-6, adiponectin, ghrelin) were measured. Participants were divided into four-groups based upon age quartile, and multivariable-adjusted least squares of means (LSM) were examined (p for trend <0.05).

**Results:**

ALT decreased with increasing age, with mean ALT levels (IU/L) of 23, 21, 20, and 17 for those between quartile ages 30–62, 63–71, 72–77, and 78–93 years (p<0.0001). Trends of decreasing LSM ALT with age and the decreasing prevalence of categorically defined elevated serum ALT with age remained robust after adjusting for sex, alcohol use, metabolic syndrome components, and biomarkers of adiposity (p-value <0.0001), and was not materially changed after adjusting for bilirubin, GGT, and albumin.

**Conclusions:**

ALT levels decrease with age in both men and women independent of metabolic syndrome components, adiposity signaling biomarkers, and other commonly used liver function tests. Further studies are needed to understand the mechanisms responsible for a decline in ALT with age, and to establish the optimal cut-point of normal ALT in the elderly.

## Introduction

Serum alanine aminotransferase (ALT) level is frequently used as a surrogate marker for hepatocyte injury. Normal ranges vary according to the commercial kit used, but have historically been set at around 40 IU/L. This value was based on population studies conducted before the availability of blood tests for hepatitis C and before the widespread recognition of nonalcoholic fatty liver disease (NAFLD). Recently, some have suggested that not only should the upper limits of normal for ALT be lowered, but that limits should differ based on sex as well [1], [2], [3]. Since then, many other factors have been implicated in differing ALT levels. Most of these factors are features related to the metabolic syndrome, including body mass index, waist-hip ratio, dyslipidemia, and glucose intolerance [4]–[13]. The relationship between ALT and age, however, remains somewhat ambiguous.

The 2005 American Gastroenterology Association Future Trends Committee report regarding the effects of aging on future trends in gastroenterology states that there is no effect of age on conventional liver function tests, including serum ALT [14]. A study of serum ALT concentrations in healthy Iranian blood donors also concluded that ALT did not correlate with age [15]. However, a few studies published since that time have reported that the prevalence of elevated (abnormal) ALT decreases with age [4], [6], [7], [8], [10], [11]. Some have found this association to be true in men, but not in women [3], [5], [16]. Most of these studies were performed using retrospective chart reviews of patients who had laboratory tests, including serum ALT, for medical reasons, in self-selected populations (such as blood donors), or in Asian populations, whose liver function may differ compared to Western populations. A recent study of community dwelling elderly men (over age 70) suggested that ALT may be a novel biomarker of aging, with levels decreasing with rising age, and low levels associating with frailty and reduced survival [17].

The largest United States population-based study of ALT levels is from the National Health and Nutrition Examination Survey (NHANES). In both the 1988–1994 [6] (elevated ALT defined as >40 U/L in men and >31 U/L in women) and the 1999–2002 [7] (elevated ALT defined as >43 U/L in both men and women) cross-sectional reports, older age was associated with a decreased prevalence of elevated ALT levels, but the conventionally higher ALT cutoff values were used in these analyses. It is therefore uncertain whether age was associated with serum ALT concentrations *within the normal range*, and whether the associations between age and the prevalence of categorically defined elevated ALT would have remained had lower ALT thresholds been applied. The latter question was answered by Ruhl and Everhart [4], who examined data from the NHANES 1988–1994 survey using both the traditionally higher ALT cutoff threshold (>43 IU/L) as well as lower thresholds (≥30 IU/L for men and ≥19 IU/L for women). They found that in univariate analysis, younger age was associated with abnormally elevated ALT activity.

Thus, there seems to be an association between younger age and increased prevalence of elevated ALT. However, it is still unclear whether serum ALT levels within the normal range decline with age, and if so, whether this decline is related to changes in body mass, central adiposity, or other plausible covariates. Additionally, it is unclear whether the threshold of extreme values of ALT (e.g. 95^th^ percentile of ALT range for a particular age group) also varies with age.

To the best of our knowledge, this paper is the first to examine the association between serum ALT level as a continuous variable and age, including ALT levels that are within normal range as well as those that are elevated, in a well-characterized [12], [18], [19], healthy, community-dwelling cohort of older men and women residing in Southern California. We also determined whether any age-related changes in serum ALT were explained by metabolic syndrome components, adiposity-related biomarkers including leptin, adiponectin, ghrelin and interleukin-6 (IL-6), or other markers of hepatic function (bilirubin, albumin, gamma glutamyl transferase [GGT]).

## Methods

### Study Cohort and Setting

This cross-sectional study is based on data from participants of the Rancho Bernardo Study (RBS). The RBS cohort was established in 1972, when 82% of residents of a geographically defined suburban Southern California community were recruited to a study of heart disease risk factors. The details of the cohort, selection criteria, and purpose of the RBS have been published elsewhere [18], [19]. The current study cohort was derived from 2480 residents who attended the research clinic between 1984 and 1987, approximately 80% of the surviving, local, community-dwelling adults. Of these, 2364 participants were aged 30 years or older and had available ALT data and were included in the current analysis [12]. The RBS cohort is almost entirely Caucasian of European ancestry, most with at least some college education, and largely white-collar workers. All participants gave written informed consent; the study was approved by the institutional review board of the University of California, San Diego.

### Clinical and Laboratory Assessment

A trained interviewer gathered information regarding medical history and current medication use. Weight was obtained with participants wearing light clothing and no shoes. Height, waist and hip circumference, and systolic blood pressure were obtained in clinic by trained investigators. Blood pressure was obtained from two morning readings with the resting participant in the seated position, using a regularly calibrated mercury sphygmomanometer, according to the Hypertension Detection and Follow-up Program protocol [20]. Alcohol use, in terms of amount, type, and frequency, was self-reported. One alcoholic drink was defined as 10 g of alcohol. Alcohol use was indirectly validated by showing a similar quantitative response to a nutritionist interviewer who obtained alcohol intake as part of a separate food-frequency questionnaire. Fasting venous blood was analyzed in a clinical laboratory. ALT, bilirubin, GGT and albumin were measured using a single assay by spectrophotometry on fresh samples. Fasting serum samples were obtained during the research visit; they were measured in all participants using the same assay and under identical testing conditions at a UCSD clinical laboratory. Total cholesterol, high-density lipoprotein (HDL) cholesterol, and triglyceride levels were measured using enzymatic methods in a Lipid Research Clinic-certified laboratory. Plasma glucose was measured in a hospital laboratory using the glucose-oxidase method. Diabetes was defined as a fasting glucose of ≥126 mg/dL (≥7 mmol/L) or treatment with either insulin or an oral hypoglycemic medication. Adipocytokine studies were performed on samples stored at −70°C. IL6 was measured using an enzyme-linked immunosorption assay (Quantikine HS, R&D Systems, Minneapolis, MN) on previously unthawed samples in 2000. Adiponectin, ghrelin, and leptin were measured in 2004 (samples thawed for a second time) by radioimmunoassay (Linco Diagnostics Laboratory, St. Louis, MO) as previously reported [21], [22]. The laboratory reports that there is no loss of assay sensitivity or adipocytokine degradation with two freeze-thaw cycles, and levels in our study are similar to those reported in the literature using the same assays [23], [24], [25], [26]. Adequate blood samples for serum leptin, IL-6, adiponectin, and ghrelin concentrations were available for 1565, 1843, 1572, and 1556 of 2364 individuals, respectively.

Elevated ALT was defined, a priori, as an ALT ≥30 IU/L for men and ≥19 IU/L for women as proposed by Prati et al [2].

### Statistical Analysis

The cohort was divided into the following four groups based upon the age quartiles: 30–62, 63–71, 72–77, and 78–93 years. Descriptive statistics were described in participants classified into these quartile age groups. Serum ALT, bilirubin, GGT, albumin, triglycerides, IL-6, leptin, adiponectin, and ghrelin were log transformed for statistical analyses to fulfill conditions of normality. Least squares means were used to examine the trends in serum ALT, bilirubin, GGT, and albumin across the four age groups and p-value for trend was examined. Multivariate hierarchical models were used to examine the relationship between ALT and age that included: 1. Un-adjusted, 2. Multiply adjusted for sex, BMI, systolic blood pressure, alcohol use, waist-hip ratio, diabetes, fasting glucose, total cholesterol-HDL cholestrol ratio, triglycerides, IL-6, leptin, adiponectin, and ghrelin, and 3. Multiply adjusted with the above plus commonly used liver function tests (bilirubin, GGT, and albumin). Additionally, we conducted multivariate-adjusted models to examine the association between age and prevalence of categorically defined elevated serum ALT across these four age groups, and p-value for trend was examined. Statistical analyses were conducted using SAS version 9.2 (SAS Institute, Cary, NC). A two-tailed p-value of less than 0.05 was considered statistically significant.

## Results

### Population Characteristics

Cohort characteristics of the 1044 men (44.2%) and 1320 women (55.8%) are shown in table 1. The mean ± standard deviation (SD) age was 69.6±10.5 years, range 30–93 years. Average (mean ± SD) BMI was 24.9±3.7 kg/m^2^ and average waist-hip ratio was 0.8±0.1. 36.8% reported no alcohol use, 22% reported <1 drink/day, 26.8% reported 1–2 drinks/day, and 14.5% reported >2 drinks/day. The mean ± SD systolic blood pressure was 139±22 mmHg. The mean ± SD for total and HDL cholesterol was 220±40 mg/dL and 62±19 mg/dL, respectively. 14.3% of the cohort had diabetes. Geometric mean (95% CI) concentrations for leptin, IL-6, adiponectin, and ghrelin were 8.6 µg/L (8.2–8.9 µg/L), 2.4 pg/ml (2.3–2.5pg/ml), 11.7 µg/ml (11.3–12.0 µg/ml), and 1345.3 pg/ml (1317.9–1373.2 pg/ml), respectively. Geometric means (95% CI) for bilirubin, GGT, and albumin were 0.5 mg/dl (0.5–0.5 mg/dl), 9.8 IU/L (9.5–10.1 IU/L), and 4.3 g/dl (4.3–4.3 g/dl), respectively.

**Table 1:** Cohort Characteristics.

	Total	Age Quartile 1 (30–62 years)	Age Quartile 2 (63–71 years)	Age Quartile 3 (72–77 years)	Age Quartile 4 (78–93 years)	P-trend
**No of individuals**	2364	597	574	589	604	
**Women (%)**	55.8	57.0	57.8	58.2	50.5	.0357
**BMI (kg/m^2^) (Mean**±**SD)**	24.9±3.7	25.5±4.0	25.2±3.7	24.7±3.6	24.4±3.4	<.0001
**Waist-hip ratio (Mean**±**SD)**	0.8±0.1	0.8±0.1	0.8±0.1	0.8±0.1	0.8±0.1	<.0001
**Alcohol use (%)**						<.0001
** None**	36.8	36.5	34.8	32.1	43.4	
** <1 drink/day**	22.0	25.0	18.8	22.6	21.4	
** 1–2 drinks/day**	26.8	22.5	27.0	31.4	26.5	
** >2 drinks/day**	14.5	16.1	19.3	13.9	8.8	
**Systolic blood pressure (mmHg) (Mean**±**SD)**	138.9±21.9	124.0±18.4	136.3±18.7	144.5±19.2	150.7±21.5	<.0001
**Total cholesterol (mg/dl) (Mean**±**SD)**	219.9±40.0	218.7±36.6	228.1±41.5	219.6±40.5	213.5±39.9	.0009
**HDL cholesterol (mg/dl) (Mean**±**SD)**	61.7±18.7	60.9±18.2	62.3±19.9	62.9±20.1	60.9±16.7	.8538
**Total/HDL cholesterol ratio (Mean**±**SD)**	3.9±1.3	3.9±1.3	4.0±1.3	3.8±1.3	3.8±1.2	.0120
† **Triglycerides (mg/dl) (95% CI)**	102.0 (99.8–104.3)	100.2 (95.9–104.7)	111.3 (106.4–116.4)	101.7 (97.3–106.3)	95.8 (91.7–100.1)	.0245
**Diabetes (%)**	14.3	7.4	12.5	17.7	19.7	<.0001
† * **Leptin (µg/L)** (**95% CI)**	8.5 (8.2–8.9)	7.1 (6.6–7.8)	9.2 (8.5–9.9)	9.1 (8.5–9.8)	8.6 (8.0–9.2)	.0015
† * **Adiponectin (µg/ml) (95% CI)**	11.7 (11.3–12.0)	8.6 (8.1–9.2)	10.7 (10.1–11.3)	12.7 (12.1–13.4)	14.0 (13.3–14.7)	<.0001
† * **Ghrelin (pg/ml) (95% CI)**	1345.2 (1317.9–1373.2)	1333.3 (1272.9–1396.5)	1303.7 (1249.5–1360.2)	1367.2 (1313.8–1422.7)	1366.8 (1316.6–1418.9)	.2028
† * **IL-6 (pg/ml) (95% CI)**	2.4 (2.3–2.5)	1.8 (1.7–1.9)	2.3 (2.2–2.5)	2.8 (2.6–3.0)	3.1 (2.9–3.3)	<.0001
**ALT (IU/L) (Mean** ± **SD)**	20.2±15.6	22.8±14.7	20.8±13.6	19.8±21.2	17.3±10.2	<.0001
** Men**	22.0±19.1	26.5±14.9	22.7±15.6	21.4±30.5	17.9±9.1	<.0001
** Women**	18.8±11.9	20.1±14.0	19.5±11.8	18.6±10.2	16.8±11.1	.0003
† **ALT (IU/L)** **(95% CI)**	17.4 (17.1–17.8)	19.6 (18.9–20.4)	18.1 (17.4–18.9)	16.9 (16.2–17.6)	15.4 (14.8–16.1)	<.0001
**Elevated ALT (%)**	28.0	35.3	31.0	28.4	17.4	<.0001
**Men (≥30 IU/L)**	15.3	28.0	15.3	11.4	7.7	<.0001
**Women (≥19 IU/L)**	38.0	40.9	42.5	40.5	26.9	.0004
† **GGT (IU/L) (95% CI)**	9.8 (9.5–10.1)	9.7 (9.1–10.2)	10.5 (9.9–11.1)	9.9 (9.3–10.4)	9.3 (8.8–9.9)	.2000
† **Bilirubin (mg/dl) (95% CI)**	0.5 (0.5–0.5)	0.4 (0.4–0.4)	0.4 (0.5–0.5)	0.5 (0.5–0.5)	0.6 (0.5–0.6)	<.0001
**Albumin (g/dl) (95% CI)**	4.3 (4.3–4.3)	4.4 (4.4–4.5)	4.4 (4.4–4.4)	4.2 (4.2–4.3)	4.1 (4.1–4.2)	<.0001

† Geometric means.

*Smaller sample sizes: Leptin (N = 1565), Adiponectin (N = 1572), Gherlin (N = 1556), IL-6 (N = 1843).

CI  =  confidence interval; SD  =  standard deviation.

Analysis of each of these variables by age showed a significant age trend for BMI, waist-hip ratio (marker of central adiposity), alcohol use, systolic blood pressure, elevated cholesterol, prevalence of diabetes, leptin, adiponectin, IL-6, bilirubin, and albumin with increasing age. BMI and albumin decreased with increasing age, while waist-hip ratio, systolic blood pressure, prevalence of diabetes, adiponectin, IL-6, and bilirubin increased with age. GGT did not show a significant trend with age.

### ALT Trends

#### Serum ALT Declines With Age

For all participants combined, ALT was highest in the youngest (30–62) age group, with a mean ± SD ALT of 23±15 IU/L for that group. Subsequently, ALT decreased with increasing age, from 21±14 IU/L for those aged 63–71 years, to 20±21 IU/L for those aged 72–77 years, and 17±10 IU/L for those aged 78–93 years.

**Figure 1:**
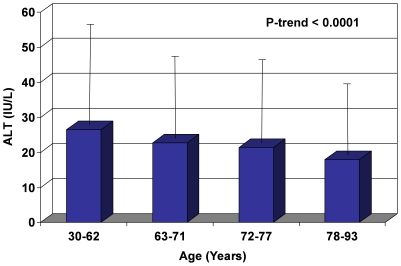
Effect of Age on ALT Values in Men (Mean and 95% Confidence Interval). In men, mean ALT (bars) and 95th percentile cutoff ranges (extension lines) both decrease with rising age.

**Figure 2:**
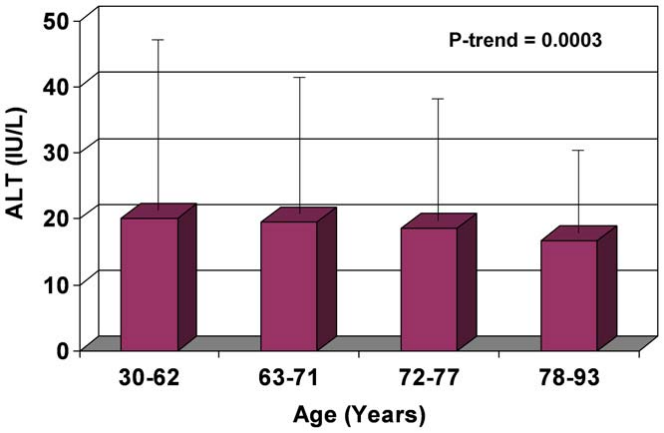
Effect of Age on ALT Values in Women (Mean and 95% Confidence Interval). In women, mean ALT (bars) and 95th percentile cutoff ranges (extension lines) both decrease with rising age.

In sex-specific analyses, ALT decreased with increasing age for both men and women. In men, the highest ALT of 26.5±14.9 IU/L was observed in the 30–62 year age group, subsequently dropping to 22.7±15.6 IU/L, 21.4±30.5 IU/L, and 17.9±9.1 IU/L for each of the progressive age subcategories (Figure 1). ALT in women started at 20.1±14.0 IU/L in the 30–62 year age group, then gradually dropped thereafter to 19.5±11.8 IU/L, 18.6±10.2 IU/L, and 16.8±11.1 IU/L for the subsequent age subcategories (Figure 2). The 95th percentile cutoff value for ALT also decreased with age, suggesting that extreme ALT values for each respective age-category declined as well (Figures 1 and 2).

#### Least Squares of Mean of ALT and Age

Results remained consistent when least squares means of serum ALT were analyzed across all four age groups, using p-value for trend (p-value <0.0001). After multivariable adjustment for sex, BMI, systolic blood pressure, alcohol use, waist-hip ratio, diabetes, fasting glucose, total cholesterol to HDL radio, triglycerides, IL-6, adiponectin, and ghrelin, the trend of decreasing least squares means of ALT with age remained consistent and statistically significant (p<0.0001). Addition of bilirubin, GGT, and albumin to the multivariable model did not significantly change the statistical association (p<0.0001) (Table 2).

**Table 2:** Least Square Means of ALT by Age.

ALT	Age Quartile 1 (30–62 years)	Age Quartile 2 (63–71 years)	Age Quartile 3 (72–77 years)	Age Quartile 4 (78–93 years)	P-trend
**Unadjusted**	19.6	18.1	16.9	15.4	<.0001
† * **Multivariate adjusted**	21.5	18.5	17.4	15.9	<.0001
† * **Multivariate adjusted with addition of GGT, bilirubin, and albumin**	20.9	18.3	17.5	16.4	<.0001

† Multi-variate adjustment for sex, BMI, systolic blood pressure, alcohol use, waist-hip ratio, diabetes, fasting glucose, total-HDL ratio, triglycerides, leptin, adiponectin, ghrelin, and IL-6.

*Smaller sample sizes: Leptin (N = 1565), Adiponectin (N = 1572), Gherlin (N = 1556), IL-6 (N = 1843).

Geometric means used for ALT, leptin, adiponectin, ghrelin, GGT, and bilirubin.

#### Elevated ALT Declines With Age

**Figure 3:**
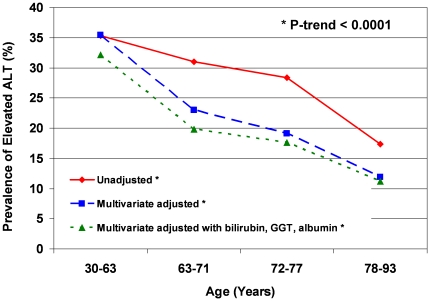
Prevalence of Elevated ALT by Age. The trend of decreasing prevalence of elevated ALT with rising age persists in unadjusted analysis, after adjustment for sex, BMI, systolic blood pressure, alcohol use, waist-hip ratio, diabetes, fasting glucose, total-HDL ratio, triglycerides, adiposity biomarkers (leptin, adiponectin, ghrelin, IL-6), and after adjustments for the above plus bilirubin, GGT, and albumin.

Using elevated ALT cutoff values of ≥30 IU/L for men and ≥19 IU/L for women as proposed by Prati et al. [2], the prevalence of abnormally elevated ALT was highest in the 30–63 year age group at 35.3%, then subsequently gradually declined to 31%, 28.4%, and 17.4% for each progressive age subcategory (p<0.0001) (Figure 3).

Similarly, after multivariate adjustment of prevalence of elevated ALT for sex, BMI, systolic blood pressure, alcohol use, waist-hip ratio, diabetes, fasting glucose, total cholesterol to HDL ratio, triglycerides, leptin, IL-6, adiponectin, ghrelin, bilirubin, GGT, and albumin, results remained statistically significant and showed a consistent downward trend of prevalence of elevated ALT with increasing age (p = 0.0001, Figure 3).

### Trends in Bilirubin, GGT, and Albumin

In unadjusted analyses, rising bilirubin (from 0.4 mg/dL in the youngest quartile to 0.6 mg/dL in the oldest quartile) and falling albumin (from 4.4 g/dL in the youngest quartile to 4.1 g/dL in the oldest quartile) were associated with increasing age, while GGT had no association. After multivariable adjustments for sex, BMI, systolic blood pressure, alcohol use, waist-hip ratio, diabetes, fasting glucose, total-HDL ratio, triglycerides, and adiposity biomarkers (leptin, adiponectin, ghrelin, IL-6), trends in rising bilirubin and falling GGT and albumin levels with increasing age remained significant with p-values for trend <0.05. Bilirubin increased from 0.4 mg/dL in the youngest group to 0.6 mg/dL in the oldest group, GGT decreased from 11.7 IU/L in the youngest group to 9.9 IU/L in the oldest group, and albumin decreased from 4.5 g/dL to 4.2 g/dL in those groups.

## Discussion

### Principal Findings

In this population-based, community-dwelling cohort of older men and women residing in Southern California, ALT levels decreased with age for both sexes. Even after multivariate adjustments for factors previously found in other studies to be associated with differing ALT levels (sex [2], [4], [7], [8], [11]; alcohol use [7]; components of the metabolic syndrome [4]–[11]: BMI, waist-hip ratio, diabetes, fasting glucose, total cholesterol to HDL ratio, triglycerides), for surrogate markers of adiposity (leptin [4], IL-6 [27], [28], adiponectin [29], [30], ghrelin [31], [32]), and for other markers of liver function (bilirubin, GGT, albumin), the association of rising age with decreasing ALT remained significant (p<0.0001, Table 2). Correspondingly, the extreme values of ALT (95^th^ percentile cutoff points for upper limit of ALT, Figures 1 and 2) as well as the prevalence of elevated ALT (p<0.0001, Figure 3) both decreased with rising age as well. These data suggest that age is an independent and robust predictor of serum ALT within normal range as well as of elevated serum ALT, and this association is independent of adiposity, known metabolic correlates of ALT, and possibly liver function. We also found that serum albumin declines with increase in age, perhaps related to a decline in muscle mass. Serum GGT did not decrease with increase in age in unadjusted analyses but after adjustment for metabolic correlates it also showed a decrease with age. Bilirubin increased with increasing age.

### Speculations on Causes and Implications for Future Research

We initially hypothesized that perhaps this trend may be related to decreasing adiposity with advancing age, leading to a decreased prevalence of nonalcoholic fatty liver disease. In analysis of our cohort characteristics, while BMI decreased with age, waist-hip ratio, and IL-6 increased with age, suggesting that central obesity (a component of the metabolic syndrome) increased with age. This finding is somewhat contrary to our hypothesis. These age-related anthropometric and cytokine changes are complex and do not account for the decline in serum ALT. Furthermore, age retained a clinically and statistically significant association with ALT despite multivariable adjustment for these metabolic characteristics and biomarkers.

Another potential explanation is that a decrease in ALT signifies a true decrease in prevalence of liver disease. While the prevalence of significant alcohol use in our cohort did decline with older age, peak consumption occurred at ages after ALT levels started to fall. Furthermore, the trend of decreasing ALT with age persisted after adjusting for alcohol intake, suggesting that alcohol consumption alone does not account for the decrease in ALT with age.

Information regarding viral hepatitis was not available in our study cohort, therefore these participants could not be excluded. Theoretically, a higher burden of chronic viral hepatitis in younger individuals could lead to higher ALT levels in this age group. However, data from NHANES suggests that this is not the case. The prevalence of hepatitis B infection was found to increase with increasing age among Caucasians [35]. While hepatitis C prevalence decreased among Caucasians aged 50 and older, the prevalence rate was only 0.7% [36], therefore any effects of decreasing hepatitis C prevalence would be offset by increasing hepatitis B prevalence, making the overall contribution of viral hepatitis to population ALT trends minimal.

Decreased prevalence of liver disease may also reflect selective survival bias, whereby those with high levels are more likely to have died. A study of 8043 construction workers by Arndt et al. [33] found that elevated liver enzymes predicted a significantly increased risk of all-cause mortality. Adams et al. [34] found that mortality was significantly higher in community-dwelling patients with NAFLD compared to the general population. Due to the cross-sectional nature of this study, we cannot exclude this possibility. Alternatively there is a yet unidentified lifestyle or clinical factor which confers protection to liver disease as one ages.

Another possibility is that decreasing ALT signifies a decrease in the mass or function of the normal liver. This is plausible given that albumin levels decreased with age and bilirubin levels increased with age. Both albumin and bilirubin provide information regarding functional status of the liver and are considered to be true liver function tests. Studies comparing young and old livers in rats showed that older livers have slower and weaker regenerative capacity, a decrease in homeostatic capacity, and a lower inflammatory response rate [37]. In rat models of liver transplantation, aged livers showed a reduced organ weight, increased hepatocyte degeneration, and increased fibrosis [38], [39]. Similarly in humans, older livers show a progressive decrease in size and blood flow, and changes related to the accumulation of oxidative stress [40], [41], [42]. In studies by Elinav et al. [43] and Le Couteur et al. [17], an ALT value below the median was a strong and independent predictor of mortality in community-dwelling elderly men. In our study, modest decreases in albumin and GGT and increases in bilirubin were observed with increasing age after adjustments for sex, alcohol use, metabolic syndrome traits, and surrogate markers of adiposity, suggesting that a decrease in liver function may indeed be present. However, bilirubin, GGT, and albumin are also complex biomarkers and may not solely reflect hepatic function. Furthermore, the trend of decreasing ALT with increasing age remained significant after adjusting for bilirubin, GGT, and albumin.

An additional possibility is that the prevalence of liver disease is the same or perhaps higher in elderly subjects, but the ALT is not a reliable marker of hepatocyte injury. In our study, age was associated with increasing central obesity, systolic blood pressure, and prevalence of diabetes, all factors associated with the metabolic syndrome and NAFLD. Although ALT is frequently used in the evaluation of NAFLD, it has been shown to correlate poorly with liver histology in subjects of all ages [44], [45]. This lack of correlation with ALT levels in the elderly could be increased in part due to a decrease in the inflammatory response of the older liver, as discussed above. Others have suggested that elderly subjects with NAFLD tend to have more fibrosis on liver biopsy compared to younger subjects who tend to have more steatosis or steatohepatitis. Frith et al. [46] examined 351 consecutive patients with biopsy-proven NAFLD, and found that age >60 was significantly associated with fibrosis. Similar results were found by Angulo et al. [47] and Miyaaki et al. [48].

Finally, ALT was a marker of age independent of other liver function tests, including bilirubin, GGT, and albumin. Others have also speculated that ALT may be a novel biomarker of aging [17].

### Strengths and Limitations

The strengths of this study include the use of a population-based, well-characterized cohort of healthy, community-dwelling elderly men and women. Analysis of data obtained from a research clinic visit 12–15 years after the initial establishment of the cohort allowed the unique opportunity to study an older group of individuals. One potential limitation of our study is that information regarding chronic liver diseases, such as viral hepatitis, autoimmune hepatitis, or metabolic liver diseases, was not available, thus we were not able to exclude these individuals. However, these conditions are not so common that they would be expected to influence the results. Our study cohort is fairly uniform (predominantly Caucasian, middle to upper-middle class), suggesting good internal validity of the findings. However, generalizability of these findings in other ethnic groups or socio-economic classes would require further studies in other more diverse cohorts.

In conclusion, ALT levels were not constant, but decreased with increasing age for both men and women, independent of components of the metabolic syndrome, surrogate markers of adiposity, and other markers of hepatic function. As a consequence, an ALT value which is considered normal for a younger individual may fall outside of 2 standard deviations for an older individual. The reasons for these differences and the clinical implications of decreasing ALT with age remain to be determined. Future longitudinal studies are needed to confirm our findings and examine the factors that explain the association between the decline in serum ALT with rising age.
